# LC‐ESI‐QTOF‐MS/MS characterization of phenolic compounds in Australian native passion fruits and their potential antioxidant activities

**DOI:** 10.1002/fsn3.3928

**Published:** 2024-01-02

**Authors:** Haoyao Liu, Osman Tuncay Agar, Ali Imran, Colin J. Barrow, Frank R. Dunshea, Hafiz A. R. Suleria

**Affiliations:** ^1^ School of Agriculture, Food and Ecosystem Sciences, Faculty of Science The University of Melbourne Parkville Victoria Australia; ^2^ Department of Pharmacognosy, Faculty of Pharmacy Suleyman Demirel University Isparta Turkey; ^3^ Department of Food Science, Faculty of Life Science Government College University Faisalabad Pakistan; ^4^ Centre for Sustainable Bioproducts, School of Life and Environmental Sciences Deakin University Waurn Ponds Victoria Australia; ^5^ Faculty of Biological Sciences The University of Leeds Leeds UK

**Keywords:** antioxidant capacity, Australian passion fruits, LC‐ESI‐QTOF‐MS/MS, phenolic profiles, polyphenolic content

## Abstract

Passion fruits, renowned globally for their polyphenolic content and associated health benefits, have enjoyed growing attention from consumers and producers alike. While global cultivar development progresses, Australia has pioneered several native cultivars tailored for its distinct planting conditions. Despite their cultivation, comprehensive studies on the phenolic profiles and antioxidant capacities of these Australian‐native passion fruits are notably lacking. This study aims to investigate and compare the polyphenolic content present in the by‐products, which are peel (L), and consumable portions, which are the pulp and seeds (P), of four indigenous cultivars: ‘Misty Gem’ (MG), ‘Flamengo’ (FG), ‘Sweetheart’ (SW), and ‘Panama’ (SH). Employing LC‐ESI‐QTOF‐MS/MS for profiling, a comprehensive list of polyphenols was curated. Additionally, various antioxidant assays—DPPH, FRAP, ABTS, RPA, FICA, and •OH‐RSA—were performed to evaluate their antioxidant potential. A total of 61 polyphenols were identified, categorized into phenolic acid (19), flavonoids (33), and other phenolic substances (9). In the antioxidant assays, the SHP sample exhibited the highest •OH^−^‐RSA activity at 98.64 ± 1.45 mg AAE/g, while the FGL sample demonstrated prominent DPPH, FRAP, and ABTS activities with values of 32.47 ± 1.92 mg TE/g, 62.50 ± 3.70 mg TE/g, and 57.84 ± 1.22 mg AAE/g, respectively. Additionally, TPC and several antioxidant assays had a significant positive correlation, including DPPH, FRAP, and ABTS. The Australian‐native passion fruits revealed distinct polyphenolic profiles and diverse antioxidant capacities, establishing a foundation for deeper health benefit analyses. This study accentuates the significance of understanding region‐specific cultivars and their potential nutraceutical applications.

## Graphical abstract

In this study, we profiled polyphenols and assessed the antioxidant potential of by‐products and consumable parts of four Australian‐native passion fruit cultivars using LC‐ESI‐QTOF‐MS/MS. We identified 61 polyphenols, revealing a rich diversity in phenolic profiles and antioxidant activities among the samples. Our findings highlight the robust antioxidant capacity of the passion fruit peels, significantly higher than that of the pulp and seeds, underscoring the nutraceutical potential of region‐specific cultivars.

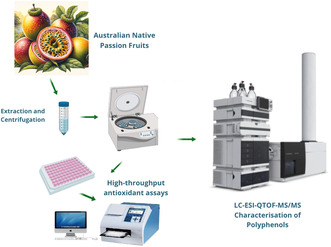

## 1 INTRODUCTION

Passion fruits, belonging to the genus *Passiflora* L., boast a diverse genetic base. While approximately 400 known species exist, a majority are cultivated for ornamental purposes, with only a subset being edible (Peña et al., 2002). These edible varieties serve as direct consumables, ingredients in dishes, or are utilized for juice production. The past few decades have witnessed a surge in the industrialization of edible passion fruit products, driven by their perceived health benefits. This has catalyzed heightened interest in the tropical fruit market, leading to a proliferation of producers. Even though South America, with nations like Brazil, Colombia, and Peru, stands as the origin epicenter of passion fruit, commercial plantations have sprouted across the globe, including locations such as Australia, Hawaii, the USA, India, South Africa, and China (He et al., 2020).

In Australia, passion fruit species became a naturalized crop in the mid‐twentieth century (Viuda‐Martos et al., 2020). The species *Passiflora* edulis Sim, introduced first, has since become the dominant commercial passion fruit. This includes two varieties: *P*. *edulis* var. *edulis* and *P*. *edulis* f. *flavicarpa*, commonly referred to as purple and yellow passion fruits, respectively, due to their distinct peel colors (Melville, 1952; Peña et al., 2002). To capitalize on the best features of both, Australian agricultural efforts have led to the development of cultivars like ‘Misty Gem’ and ‘Sweetheart’. Recent advancements have given rise to newer cultivars, such as ‘Flamingo’ (Kretzschmar, 2020). In Australia, passion fruit species became a naturalized crop in the mid‐twentieth century. The annual production of passion fruit in Australia is approximately 4790 tons, valued at around $16.8 million, with the majority sold as fresh fruit and about 302 tons processed, as reported by Horticulture Australia (2003). Despite the industry's size, there is a noticeable gap regarding Australian native passion fruits and their phytochemical content, indicating a significant area for research and development within the sector.

Tropical fruits, including passion fruits, are the focus of many studies investigating their potential antioxidant abilities. It is now understood that free radicals contribute to the damage of lipids, proteins, and nucleic acids, leading to a range of health issues such as inflammation, aging, diabetes, cardiovascular diseases, and other chronic illnesses (Contreras‐Calderón et al., 2011). Phytochemicals, including vitamins A and C, as well as phenolic acids, have been reported to have the ability to scavenge free radicals and alleviate cellular oxidative stress (Contreras‐Calderón et al., 2011; Juliana Kelly da Silva et al., 2014).

Phenolic compounds in plants are particularly noteworthy, courtesy of their myriad health benefits. Passion fruits have been flagged as significant phenolic compound reservoirs, holding their own against other phenolic‐rich fruits like citrus (Septembre‐Malaterre et al., 2016). Some studies posit that fruit by‐products, for instance, peels and seeds, are more densely packed with phenolic compounds than their edible counterparts. Given that almost 40% of passion fruit output feeds the juice industry, understanding these by‐products becomes paramount.

Our study aims to establish the antioxidant potential and characterize the phenolic compound profiles of four native Australian passion fruit cultivars. The insights gained from this research are anticipated to contribute to the advancement of both the fruit industry and future medicinal investigations.

## 2 MATERIALS AND METHODS

### 2.1 Chemicals and reagents

This investigation utilized analytical‐grade chemicals. Sigma‐Aldrich Corporation (Castle Hill, NSW, Australia) was the primary supplier, providing substances that included aluminum chloride hexahydrate, potassium persulfate, and the Folin–Ciocalteu's phenol reagent. Additionally, the free radical scavengers 2,2‐diphenyl‐1‐picrylhydrazyl (DPPH) and 2,4,6‐tripyridyl‐s‐triazine (TPTZ), along with 2,20‐azino‐bis‐(3‐ethylbenzothiazoline‐6‐sulfonic acid), and a suite of standard compounds such as quercetin, vanillin, and catechin, were acquired. Supplies of acetic acid, ethanol, sulfuric acid, sodium acetate, sodium carbonate, and ferric chloride hexahydrate (FeCl₃·6H₂O) were procured from Thermo Fisher Scientific (Scoresby, Melbourne, VIC, Australia). Sigma‐Aldrich (St. Louis, MO, USA) provided additional standards, which encompassed protocatechuic acid, syringic acid, chlorogenic acid, caftaric acid, caffeic acid, p‐hydroxybenzoic acid, coumaric acid, gallic acid, epicatechin gallate, catechin, kaempferol, quercetin‐3‐galactoside, kaempferol‐3‐*O*‐glucoside, quercetin‐3‐*O*‐glucuronide, and quercetin standards.

### 2.2 Sample preparation

Approximately 20–30 fruits from each of the four passion fruit cultivars (Flamingo, Misty Gem, Sweetheart, and Panama), weighing a total of two to three kilograms, were obtained. These cultivars were sourced from an Australian local fruit trader, ‘Ten Farms Fruits Trader’. After cleaning and separation, each cultivar was labeled as Flamingo (FG), Misty Gem (MG), Sweetheart (SW), and Panama (SH). The fruits were then segmented into their respective parts: the edible portion, consisting of the pulp and seeds (P), and the by‐products, primarily the peel (L). All samples underwent lyophilization, were ground into fine particles, and were stored at −40°C, ensuring minimal exposure to light and air. For each cultivar, an extract was prepared from this composite of fruits. Each extract was then used to perform the experiments, with each set of experiments conducted in triplicate to ensure reproducibility and reliability.

### 2.3 Extraction of phenolic compounds

Each sample, weighing 1 gram, was subjected to extraction with 10 milliliters of 70% ethanol. The mixture was then subjected to homogenization using a T25 Ultra‐Turrax device (IKA, Staufen, Germany) set to 12,000 *g* for a duration of 30 s. Following homogenization, the samples were placed in a ZWYR‐240 incubation unit (Labwit, Ashwood, VIC, Australia) and agitated at a speed of 120 rpm for a period of 12 h. After the incubation phase, centrifugation was performed at a velocity of 10,800 rpm and a temperature of 4°C for a quarter of an hour. The supernatant was then carefully collected, appropriately labeled, and preserved at a temperature of −18°C for subsequent analytical procedures.

### 2.4 Estimation of phenolic compounds and antioxidant assays

Three assays—TPC, TFC, and TCT—were designed for phenolic compound estimation. To determine antioxidant capacity, seven assays were employed, founded on three principles: ABTS, DPPH, •OH‐RSA, and PMA, which operate on the hydrogen atom transfer principle; as well as FRAP and RPA, which function based on single electron evaluation. Additionally, the FICA assay was utilized to measure metal chelation capacity. The seven assays were performed in triplicate, following the methodology outlined by Suleria et al. (2020), with certain modifications incorporated. In these assays, ethanol was employed as the solvent of choice for dissolving the standards, including Trolox, catechin, gallic acid, and quercetin, ensuring consistent and reliable preparation across the experiments. The outcomes were subsequently quantified using the Multiskan® Go microplate reader (Thermo Fisher Scientific, Waltham, MA, USA).

#### 2.4.1 Determination of total phenolic content (TPC)

The total phenolic content assays used in this study were modified based on the Folin–Ciocalteu technique (Singleton et al., 1999). 25 μL of the extracts were added to the 96‐well plate, followed by the addition of 25 μL of Folin reagent and 200 μL of deionized water. The plate was then incubated for 5 min at room temperature. Subsequently, 25 μL of a 10% solution of sodium carbonate was introduced to each sample well. The plate then underwent a further incubation of 60 min to allow for reaction completion before the absorbance was gauged at 765 nm using a Thermo Fisher Scientific spectrophotometer (Waltham, MA, USA). For quantification purposes, a standard curve of gallic acid was utilized, with concentrations spanning from 0 to 1000 μg/mL, expressing the phenolic content as milligrams of gallic acid equivalents (GAE) per gram of the sample's dry weight.

#### 2.4.2 Determination of Total flavonoid content (TFC)

The total flavanol content estimation assay was based on the aluminum chloride method from a previous study (Peng et al., 2019). In this study, 80 μL of extract was added to the 96‐well plates, followed by the addition of 80 μL of 2% aluminum chloride solution and 120 μL of 50 g/L sodium acetate. The mixtures were incubated for 2.5 h at room temperature, and the absorbance was then measured at 440 nm. The TFC values were determined using the quercetin standard curve (ranging from 0 to 50 μg/mL) and expressed as milligrams of quercetin equivalents (QE) per gram of the dry sample weight.

#### 2.4.3 Determination of total condensed tannin content (TCT)

The assay for total condensed tannin content was based on a modified vanillin and *p*‐dimethylaminocinnamaldehyde method (Megat Rusydi & Azrina, 2012). In this study, 25 μL of passion fruit extract was added to the 96‐well plate, followed by the addition of 150 μL of a 4% vanillin reagent and 25 μL of concentrated (32%) sulfuric acid. The reaction mixture was allowed to incubate at room temperature for 25 min, after which the absorbance at 500 nm was recorded. The standard curve for this assay was constructed using a 0–1000 μg/mL catechin methanolic solution. A catechin methanolic solution was used to establish a standard curve, ranging from 0 to 1000 μg/mL, to facilitate the calculation of total condensed tannins. The results were quantified and reported as catechin equivalents (CE), in milligrams, per gram of the dried sample's weight.

#### 2.4.4 2,2‐Diphenyl‐1‐Picrylhydrazyl (DPPH) antioxidant assay

The DPPH radical‐scavenging activity was based on a modified version of the previous methods by (Siddhuraju & Manian, 2007). Initially, 25 μL of passion fruit samples were added to a 96‐well plate, followed by the addition of 275 μL of DPPH methanolic solution. The plate was then incubated for 30 min at room temperature, and the absorbance was measured at 517 nm. The standard curve for this assay was constructed using a 0–200 μg/mL Trolox aqueous solution. The DPPH values were determined using the Trolox standard curve (ranging from 0 to 200 μg/mL) and expressed as milligrams of Trolox equivalents (TE) per gram of the dry sample weight.

#### 2.4.5 Ferric‐reducing antioxidant power (FRAP) assay

In this study, the ferric‐reducing antioxidant power (FRAP) assay was adapted from (Zheng et al., 2019), which evaluates a substance's ability to reduce ferric‐2,4,6‐tripyridyl‐s‐triazine (Fe^3+^‐TPTZ) to its ferrous form (Fe^2+^‐TPTZ). To prepare the FRAP reagent, a ferric chloride (FeCl₃) solution (20 mM), a TPTZ solution (10 mM), and a sodium acetate buffer (300 mM) were combined in a volumetric ratio of 1:1:10. 20 μL of passion fruit samples were added to a 96‐well plate, followed by the addition of 280 μL of the prepared FRAP solution. The reaction was allowed to proceed at 37°C for a duration of 10 min. Post‐incubation, the reaction mixture's absorbance was measured at 593 nm. The FRAP values were determined using the Trolox calibration curve (ranging from 0 to 50 μg/mL) and expressed as milligrams of Trolox equivalents (TE) per gram of the dry sample weight.

#### 2.4.6 2,2′‐Azino‐bis‐3ethylbenzothiazoline‐6‐sulfonic acid (ABTS) radical scavenging assay

The ABTS scavenging ability estimation essay was based on a modified method of the ABTS^+^ radical cation decolorization test (Krosnick et al., 2009; Rajurkar & Hande, 2011). To form ABTS radical cations, a 7 mM ABTS stock solution (5 mL) was reacted with potassium persulfate (140 mM, 88 μL) and allowed to stand in darkness for 16 h. This ABTS+ stock was then diluted with ethanol until the absorbance reached 0.70 ± 0.02 at a wavelength of 734 nm. Subsequently, 10 μL of passion fruit samples were mixed with 290 μL of the prepared ABTS+ solution in a 96‐well plate and incubated in the dark for 6 min at 25°C. The absorbance was measured at 734 nm. The antioxidant capacity of passion fruit samples was determined using the calibration curve developed with a 0–200 μg/mL ascorbic acid aqueous solution. The ABTS values were determined using the Trolox standard curve and expressed as milligrams of Trolox equivalents (TE) per gram of the dry sample weight.

#### 2.4.7 Hydroxyl radical scavenging activity (•OH^−^‐RSA)

This assay was based on the Fenton‐type reaction approach with some modifications (Krosnick et al., 2009; Smirnoff & Cumbes, 1989). 50 μL of passion fruit supernatant was added to a 96‐well plate, followed by the addition of 50 μL of 6 mM FeSO4.7H_2_O and 50 μL of 6 mM H_2_O_2_. After 10 min of incubation at room temperature, 50 μL of 6 mM 3‐hydroxybenzoic acid was added. The plate was then incubated for an additional 10 min at room temperature, and the absorbance of the samples was measured at 510 nm. The •OH^−^‐RSA values were determined using the Trolox standard curve (ranging from 0 to 400 μg/mL) and expressed as milligrams of Trolox equivalents (TE) per gram of the dry sample weight.

#### 2.4.8 Ferrous ion‐chelating activity (FICA)

The ferrous ion‐chelating activity (FICA) in this study was determined by modifying the method from a previous study (Dinis et al., 1994). 15 μL of extracted supernatant was added to a plate, followed by the addition of 85 μL of water, 50 μL of reagent A, and 50 μL of reagent B. Reagent A was prepared by diluting FeCl_2_, while reagent B was prepared by diluting Ferrozine. After being incubated at room temperature for 10 min, the absorbance was measured at 562 nm. The FICA values were determined using the EDTA standard curve (ranging from 0 to 50 μg/mL of EDTA dissolved in NaOH with a ratio of 10 mL of 1 mg/mL EDTA to 0.022 g NaOH) and expressed as milligrams of EDTA equivalents (EE) per gram of the dry sample weight.

#### 2.4.9 Reducing power assay (RPA)

The reducing power activity was detected by modifying the method from a previous study (Ferreira et al., 2007). 10 μL of passion fruit supernatant was added to a plate, followed by the addition of 25 μL of buffer C and 25 μL of K3[Fe(CN)6]. This was followed by an incubation process at room temperature lasting for 20 min. Subsequently, 25 μL of TCA, 85 μL of water, and 8.5 μL of 0.1% (w/v) FeCl3 were added to the plate. The mixture was further incubated at room temperature, and the absorbance was measured at 750 nm. The reducing power activity values were determined using the Trolox standard curve (ranging from 0 to 500 μg/mL) and expressed as milligrams of Trolox equivalents (TE) per gram of the dry sample weight.

#### 2.4.10 Total antioxidant capacity

The total antioxidant capacity was assessed using the phosphomolybdate method (PMA), as outlined in the work of (Prieto et al., 1999). To create the phosphomolybdate reagent, 0.028 M sodium phosphate, 0.6 M sulfuric acid, and 0.004 M ammonium molybdate were combined. Subsequently, 40 μL of the extract was mixed with 260 μL of the phosphomolybdate reagent and added to a 96‐well plate. The reaction mixture was incubated at 90°C for 90 min. After cooling to room temperature, the absorbance was measured at 695 nm. The total antioxidant capacity (TAC) was determined using the Trolox standard curve (ranging from 0 to 200 μg/mL) and expressed as milligrams of Trolox equivalents (TE) per gram of the dry sample weight.

#### 2.4.11 Relative antioxidant capacity index (RACI)

The relative antioxidant capacity index (RACI) was calculated as the mean value of standardized scores from the various antioxidant assays conducted for each sample. This method, proposed by Sun and Tanumihardjo (2007), allows for an integrated evaluation of antioxidant capacity across different units of measurement and types of distribution. The standard scores for each cultivar in each assay were determined using the formula:

$$\text{standard score}=\frac{\left(x-\mu \right)}{\sigma }$$

where *x* represents the antioxidant value, *μ* is the mean of the antioxidant values across assays, and *σ* is the standard deviation. The mean of all standard scores for each cultivar was then defined as the RACI value of that cultivar.

### 2.5 LC‐ESI‐QTOF‐MS/MS analysis

The profiling of phenolic compounds was adapted from protocols developed by Zhong et al. (2020), incorporating minor adjustments, and executed on an Agilent 1200 series high‐performance liquid chromatography system linked to an Agilent 6520 Accurate Mass Quadrupole Time‐of‐Flight Liquid Chromatography‐Mass Spectrometry/Mass Spectrometry platform (Agilent Technologies, CA, USA). Separation of compounds was conducted using a Synergi Hydro‐RP 80A LC Column (250 mm × 4.6 nm, 4 μm) (Phenomenex, Torrance, CA, USA), with a Phenomenex C18 ODS (4.0 × 2.0 mm) guard column employed for column protection.

Mobile phase A was prepared by mixing water and acetic acid in a ratio of 99.8:0.2 (v/v), while mobile phase B was composed of acetonitrile, water, and acetic acid in a ratio of 50:49.8:0.2 (v/v/v). The mobile phases were subjected to degassing at a temperature of 25°C for a duration of 15 min. A gradient program was implemented, utilizing a combination of mobile phases A and B in varying proportions: starting with 90% A and 10% B at the initial timepoint (0 min), changing to 75% A and 25% B at 20 min, adjusting to 65% A and 35% B at 30 min, then to 60% A and 40% B at 40 min, followed by 45% A and 55% B at 70 min, altering to 20% A and 80% B at 75 min, switching to 100% B at 77 min, and finally reverting back to 90% A and 10% B at 85 min. The flow rate of this system was maintained at 0.8 mL/min, and the volume of the sample injected was set at 5 μL.

For peak identification, dual‐mode ionization was employed, utilizing negative and positive ion modes. Nitrogen served as both the nebulizer and drying agent, supplied at 45 psi and warmed to a temperature of 300°C at a rate of 5 L per minute. The voltage settings for the capillary and nozzle were fixed at 3.5 kilovolts and 500 volts, respectively. The mass spectra acquisition spanned from 50 to 1300 atomic mass units, with fragmentation induced by collision energies of 10, 15, and 30 electron volts. The resulting data were processed and analyzed using the Mass Hunter Data Acquisition Software (version B.03.01) from Agilent Technologies (Santa Clara, CA, USA).

### 2.6 Statistical analysis

Results for polyphenolic content and antioxidant activities are presented as mean values with accompanying standard deviations, with each study replicated three times (*n* = 3). Statistical analysis was undertaken using Minitab software (version 18.0 for Windows), applying a one‐way ANOVA and post hoc comparison via the Tukey's HSD test, with statistical significance determined at *p* < .05 (Minitab, LLC, State College, PA, USA).

## 3 RESULT AND DISCUSSION

### 3.1 Phenolic compound estimation (TPC, TFC, TCT)

In the current study, significant differences were observed in TPC, TFC, and TCT across various passion fruit varieties and portions (Table 1). The highest TPC was recorded in FGL (40.63 ± 1.86 mg GAE/g), followed by MGL (21.82 ± 0.43 mg GAE/g), SWL (17.45 ± 0.28 mg GAE/g), and SHL (15.52 ± 0.28 mg GAE/g). It is important to highlight that all the peel samples mentioned earlier displayed higher total phenolic compound (TPC) values in comparison to the pulp and seed samples. The lowest TPC was observed in SHP (3.44 ± 0.28 mg GAE/g). These findings align with previous studies that have underscored the relatively higher TPC in peels compared to pulp (Fonseca et al., 2022). Dos Reis et al. (2018) also reported a higher TPC in purple passion fruit than in yellow passion fruit. This is consistent with our findings, where MG, FG, and SH showed higher TPC values in the same portions compared to SW. Despite the Folin–Ciocalteu method being widely used for estimating polyphenol content, it can react with various reducing agents, including polyphenols, ascorbic acid, and carotenoids. Given that passion fruits are a rich source of both ascorbic acid and carotenoids, these constituents might account for the elevated TPC values. Additionally, factors like cultivation practices, maturity stages, and extraction methods can influence TPC values.

**TABLE 1:** Phenolic compound content in investigated passion fruits.

	TPC (mg GAE/g)	TFC (mg QE/g)	TCT (mg CE/g)
FGL	40.63 ± 1.86^a^	1.75 ± 0.06^a^	46.050 ± 0.20^b^
FGP	9.27 ± 0.53^e^	0.19 ± 0.02^bc^	0.76 ± 0.01^e^
MGL	21.82 ± 0.43^b^	1.74 ± 0.13^a^	55.089 ± 0.91^a^
MGP	10.37 ± 0.49^e^	0.18 ± 0.01^bc^	1.23 ± 0.07^e^
SWL	17.45 ± 0.28^c^	1.64 ± 0.12^a^	34.66 ± 0.65^c^
SWP	12.71 ± 0.41^d^	0.32 ± 0.01^b^	1.22 ± 0.08^e^
SHL	15.52 ± 0.28^c^	1.65 ± 0.09^a^	27.54 ± 0.52^d^
SHP	3.44 ± 0.28^f^	0.02 ± 0.01^c^	0.56 ± 0.03^e^

*Note*: Values are expressed as means ± SD per gram of powder weight; *n* = 3 samples per variety. Mean values within a column that have different superscript letters (a–f) differ significantly from one another (*p* < .05).

Abbreviations: CE, Catechin Equivalents; GAE, Gallic Acid Equivalents; QE, Quercetin Equivalents; TCT, Total Condensed Tannins; TFC, Total Flavonoid Contents; TPC, Total Phenolic Contents.

A similar trend between the peel and the edible portions of passion fruits was also evident for TFC. Peel samples showed markedly higher values. However, no significant differences were observed among the cultivars. The TFC values ranged between 1.64 ± 0.12–1.74 ± 0.13 mg QE/g for by‐product portions and 0.02 ± 0.01–0.32 ± 0.01 mg QE/g for edible portions.

For TCT values, peels outperformed the pulp and seeds by a significant margin. The descending order of TCT values was MGL > FGL > SWL > SHL for peels, followed by MGP, FGP, SWP, and SHP. Tannins, known to be a group of plant pigments, are responsible for the diverse colors observed in plants. The relatively elevated TCT in peel samples could be the primary reason for the darker hue of passion fruit peels (He et al., 2020).

### 3.2 Antioxidant activity estimation

In this study, assays such as DPPH, FRAP, ABTS, RPA, FICA, •OH^—^RSA, and PMA were employed to estimate the in‐vitro antioxidant activities (Table 2).

**TABLE 2:** Result of the antioxidant assays in investigated passion fruits.

	DPPH (mg TE/g)	FRAP (mg TE/g)	ABTS (mg AAE/g)	•OH^—^RSA (mg AAE/g)	PMA (mg TE/g)	RPA (mg EDTA/g)	FICA (mg AAE/g)
FGL	32.47 ± 1.92^a^	62.50 ± 3.70^a^	57.84 ± 1.22^a^	9.75 ± 0.60^e^	9.32 ± 0.21^a^	25.67 ± 2.53^c^	0.56 ± 0.01^b^
FGP	7.64 ± 0.15^d^	7.89 ± 0.71^de^	10.16 ± 0.46^d^	95.54 ± 0.69^b^	4.31 ± 0.24^c^	7.09 ± 0.38^de^	0.36 ± 0.01^c^
MGL	16.31 ± 1.02^b^	37.71 ± 2.25^b^	48.79 ± 1.86^b^	19.93 ± 1.38^c^	9.41 ± 0.25^a^	44.85 ± 2.00^a^	0.15 ± 0.01^e^
MGP	9.5 ± 0.51^cd^	9.43 ± 0.10^d^	9.82 ± 0.36^d^	95.89 ± 0.67^ab^	3.67 ± 0.15^cd^	6.85 ± 0.17^de^	0.16 ± 0.01^e^
SWL	18.2 ± 0.12^b^	22.97 ± 1.21^c^	22.10 ± 2.11^c^	19.61 ± 1.33^c^	9.43 ± 0.19^a^	32.85 ± 2.03^b^	0.72 ± 0.01^a^
SWP	11.14 ± 0.57^c^	11.14 ± 0.48^d^	10.78 ± 0.65^d^	96.11 ± 1.01^ab^	5.49 ± 0.68^b^	10.27 ± 0.98^d^	0.29 ± 0.01^d^
SHL	16.58 ± 1.37^b^	20.14 ± 1.15^c^	20.74 ± 0.79^c^	16.41 ± 0.42^d^	9.87 ± 0.14^a^	23.82 ± 1.75^c^	0.26 ± 0.02^d^
SHP	6.98 ± 0.54^d^	4.51 ± 0.30^e^	7.88 ± 0.17^d^	98.64 ± 1.45^a^	3.34 ± 0.19^d^	4.47 ± 0.33^e^	0.25 ± 0.03^d^

*Note*: Values are means ± SD per gram powder weight; *n* = 3 samples per sample. Mean values within a column with different superscript letters (a–e) are significantly different (*p* < .05).

Abbreviations: ^•^OH‐RSA, Hydroxyl radical scavenging activity assay; AAE, L‐ ascorbic acid equivalents; ABTS, 2,2′‐azino‐bis‐3‐ethylbenzothiazoline‐6‐sulfonic acid assay; DPPH, 2,2′ ‐diphenyl‐1‐picrylhydrazyl assay; FICA, Ferrous ion ‐chelating ability; FRAP, ferric reducing antioxidant power assay; GAE, gallic acid equivalents; PMA, Total antioxidant assay; RPA, Reducing power assay; TE, Trolox equivalents.

Both the DPPH and ABTS assays operate on similar principles, aiming to determine the scavenging ability against free radicals. Their values exhibited a consistent trend, with peels consistently showing higher values than their edible counterparts, which aligns with findings from Dos Reis et al. (2018). Among the cultivars, FGL exhibited the highest DPPH value (32.47 ± 1.92 mg TE/g), followed by SWL (18.2 ± 0.12 mg TE/g), SHL (16.58 ± 1.37 mg TE/g), and MGL (16.31 ± 1.02 mg TE/g). Similarly, ABTS values were highest in FGL (57.84 ± 1.22 mg AAE/g) and lowest in SHL (20.74 ± 0.79 mg AAE/g). However, both ABTS and DPPH assays have their biases: ABTS is more amenable to both hydrophilic and lipophilic chemicals, while DPPH is especially sensitive to hydrophobic molecules. Therefore, to comprehensively understand antioxidant potential, a broader array of assays is recommended. In contrast, the study by da Silva et al. (2013) presented DPPH and ABTS values for the extract aqueous of *Passiflora edulis* leaves as 1100 μg/mL and 192.2 ± 0.50 μmol TE g^−1^, respectively. While the units of measurement and the methodologies might differ between the two studies, the substantial DPPH and ABTS values in da Silva et al.'s work highlight the strong antioxidant potential inherent in the *P*. *edulis* leaves. Comparatively, the results emphasize the diverse antioxidant potential across different plant parts and species (da Silva et al., 2013).

In the •OH^−^‐RSA assay, the •OH^−^ serves as the free radical, indicative of oxidative stress in the human body. This assay assesses the scavenging ability against hydroxy‐free radicals. Interestingly, the edible portions showcased superior scavenging ability compared to peels, with values ranging from 95.54 ± 0.69 mg AAE/g to 98.64 ± 1.45 mg AAE/g.

The antioxidant assay RPA is based on the reaction between reduction‐potential substances in extracts and ferricyanide. This reaction leads to the reduction of ferricyanide (Fe^3+^) and the formation of ferrocyanide (Fe^2+^), indicating the hydrogen‐donating capacity of the tested extracts. In this study, peel samples exhibited significantly higher values than their edible counterparts. The highest RPA was found in MGL (44.85 ± 2.00 mg EDTA/g), followed by SWL (32.85 ± 2.03 mg EDTA/g), FGL (25.67 ± 2.53 mg EDTA/g), and SHL (23.82 ± 1.75 mg EDTA/g). However, RPA is sensitive to the presence of specific substances, such as proteins. Due to the complexity of plant polyphenols, RPA alone is insufficient to estimate the reducing ability of extracts. The FRAP assay is another test for reducing ability based on the reduction of ferric‐tripyridyl triazine and the formation of the ferrous complex. In these samples, the same trend of pulp samples, with the order SWP > MGP=FGP > SHP, was found in both RPA and FRAP. However, in peel samples, different FRAP values were observed, ranging from 62.50 ± 3.70 mg TE/g to 20.14 ± 1.15 mg TE/g. This difference could be attributed to the more complex composition of polyphenols between portions, resulting in the disparity between RPA and FRAP.

The PMA assay estimates the tested antioxidants' reducing capacity by measuring the reduction of molybdenum to molybdenum. The assay revealed that there was no significant difference in the reducing power among the peel samples tested: SHL (9.87 ± 0.14 mg TE/g), SWL (9.43 ± 0.19 mg TE/g), MGL (9.41 ± 0.25 mg TE/g), and FGL (9.32 ± 0.21 mg TE/g). The SWP exhibited the best PMA (5.49 ± 0.68 mg TE/g), followed by MGP (4.31 ± 0.24 mg TE/g), FGP (3.67 ± 0.15 mg TE/g), and SHP (3.34 ± 0.19 mg TE/g).

The FICA assay is designed to determine the chelating ability of the investigated samples against transition metal ions. An observation worth noting is that for the FICA results, the values of MGL (0.15 ± 0.01 EDTA/g) and SHL (0.26 ± 0.02 EDTA/g) were close to those of MGP (0.16 ± 0.01 EDTA/g) and SHP (0.25 ± 0.03 EDTA/g), respectively. This close proximity in values may be attributed to several factors. For one, both the peel and edible parts of the same variety of passion fruit may possess similar profiles of certain chelating compounds. Additionally, factors like the maturity stage of the fruits, growing conditions, and post‐harvest processing can influence the chelating ability in both parts. It is also possible that certain chelating agents present in the peel could have migrated to the edible portion, leading to comparable FICA results. It is crucial to note that while these results are unexpected, they reiterate the complexity of antioxidant activity in natural products and highlight the necessity of conducting multiple assays to gain a comprehensive understanding. We suggest further research on the migration of chelating agents between the peel and the edible part, as well as more detailed profiling of the chelating compounds present in these samples, to better understand these observations. In our study, the highest metal‐chelating ability was observed in SWL (0.72 ± 0.01 EDTA/g), while the least chelating ability was exhibited by MGL (0.15 ± 0.01 EDTA/g).

### 3.3 Relative antioxidant capacity index

**FIGURE 1:**
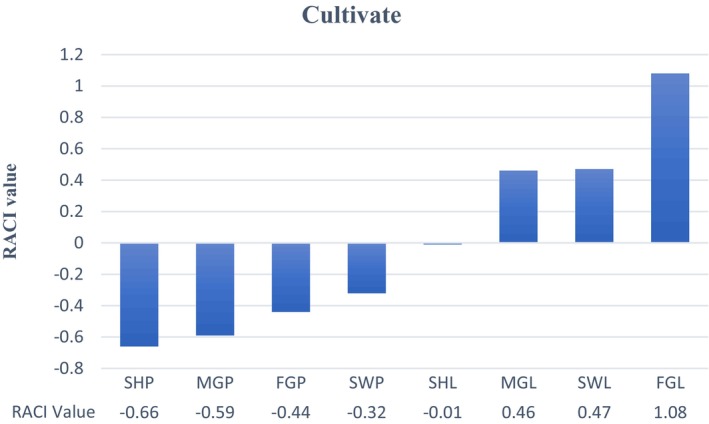
Relative antioxidant capacity index of passionfruit cultivates.

The RACI integrates different antioxidant assays to rank the antioxidant capacities of samples more effectively, irrespective of their measurement units and distribution types. As depicted in Figure 1, the cultivar ‘Flamingo Peel’ (FGL) exhibited the highest RACI value (1.08), followed by ‘Sweetheart Peel’ (SWL) at 0.47 and ‘Misty Gem Peel’ (MGL) at 0.46. Conversely, the lowest RACI values were observed in ‘Sweetheart Pulp’ (SHP) at −0.66 and ‘Misty Gem Pulp’ (MGP) at −0.59. Collectively, these RACI values suggest that the by‐products, such as the peel, demonstrate substantially higher antioxidant capacities than the consumable portions, namely the pulp and seeds.

### 3.4 Correlation analysis

**FIGURE 2:**
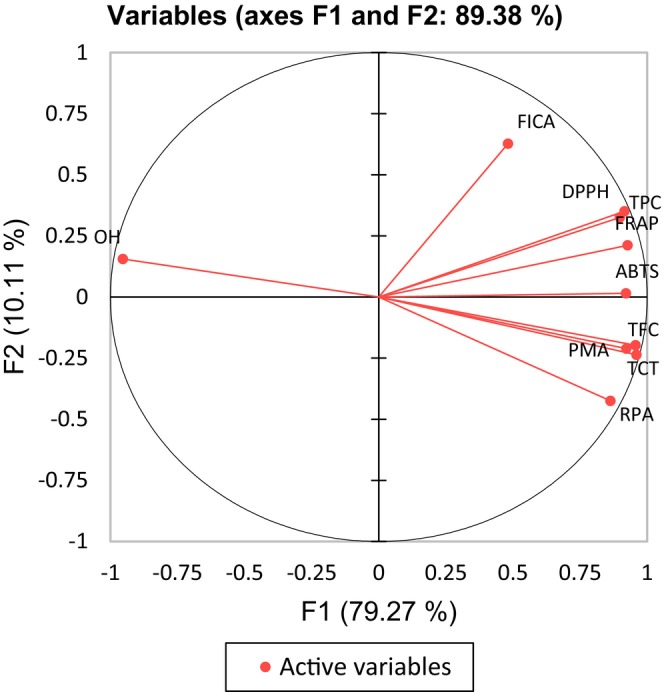
Principal components analysis (PCA) of antioxidant assays and phenolic content in investigated passion fruits.

The correlation between antioxidant assays and phenolic content was examined utilizing Pearson's correlation test, with further insights garnered through the principal component analysis (PCA). The PCA explained 89.38% of the total variance, where the first component (F1) accounted for 79.27% and the second component (F2) covered 10.11% (Figure 2).

From the PCA biplot, two distinct clusters can be identified. The first cluster groups TFC, PMA, and TCT closely together. This suggests a strong interrelationship between flavonoids, proanthocyanidins, and total condensed tannins in determining the antioxidant properties of passion fruit. The second cluster encompasses DPPH, FRAP, ABTS, RPA, and TPC, indicating that these variables have a strong linear relationship and share similar variance.

It is noteworthy that the –OH radical scavenging is located in the first quadrant of the PCA cycle. This suggests that while it has a positive relationship with the F1 component, it possesses a contrasting profile to the majority of antioxidant assays and phenolic content variables. The position of –OH radical scavenging could be a reflection of its unique mechanism of action or the specificity of the assay method.

Table 3 displays the correlations between phenolic content and antioxidant estimation assays, with significance levels marked with asterisks. Strong correlations are observed among TPC, TFC, and TCT (*r* = .750 and 0.781, *p* < .05). This underscores that a significant portion of the total phenolic content correlates with flavonoids and condensed tannins. However, it is crucial to note from Table 1 that TCT values consistently surpass TPC values, indicating that not all condensed tannins are part of the TPC. This observation aligns with the research by Dos Reis et al. (2018).

**TABLE 3:** Correlation between phenolic contents and antioxidant assays.

Variables	TPC	TFC	TCT	DPPH	FRAP	ABTS	^•^OH‐RSA	PMA	RPA	FICA
TPC	1.000	0.750^a^	0.781^a^	0.972^b^	0.982^b^	0.920^b^	−0.751	0.705	0.598	0.468
TFC	0.750^a^	1.000	0.946^b^	0.801^a^	0.786^a^	0.797^a^	−0.994	0.987^b^	0.910^b^	0.427
TCT	0.781^a^	0.946^b^	1.000	0.781^a^	0.853^b^	0.911^b^	−0.935	0.900^b^	0.951^b^	0.320
DPPH	0.972^b^	0.801^a^	0.781^a^	1.000	0.954^b^	0.867^b^	−0.817	0.763^a^	0.596	0.596
FRAP	0.982^b^	0.786^a^	0.853^b^	0.954^b^	1.000	0.973^b^	−0.790	0.727^a^	0.674	0.403
ABTS	0.920^b^	0.797^b^	0.911^b^	0.867^b^	0.973^b^	1.000	−0.792	0.732^a^	0.766^a^	0.265
^•^OH‐RSA	−0.751	−0.994	−0.935	−0.817	−0.790	−0.792	1.000	−0.974	−0.877	−0.452
PMA	0.705	0.987^b^	0.900^b^	0.763^a^	0.727^a^	0.732^a^	−0.974	1.000	0.887^b^	0.433
RPA	0.598	0.910^b^	0.951^b^	0.596	0.674	0.766^a^	−0.877	0.887^b^	1.000	0.264
FICA	0.468	0.427	0.320	0.596	0.403	0.265	−0.452	0.433	0.264	1.000

^a^ Significant correlation with *p* ≤ .05.

^b^ Significant correlation with *p* ≤ .01.

PMA also showed significant correlations with the antioxidant assays DPPH, FRAP, ABTS, and RPA. The pronounced correlations between TFC and PMA (0.987, *p* < .01) and TCT and PMA (0.900, *p* < .01) bolster the idea that flavonoids and condensed tannins majorly contribute to the antioxidant capacity of passion fruit.

Furthermore, TPC, TFC, and TCT exhibited robust correlations with the antioxidant assays. Particularly, TPC had correlations of *r* = .972, 0.982, and 0.920 with DPPH, FRAP, and ABTS, respectively (*p* < .01). For TFC, the coefficients were 0.801, 0.786, 0.797, 0.987, and 0.910 with the assays (*p* < .05), and for TCT, they were 0.781, 0.853, 0.911, 0.900, and 0.95 (*p* < .05). These data emphasize the pivotal role of flavonoids and condensed tannins in the antioxidant potential of passion fruit.

### 3.5 LC‐ESI‐QTOF‐MS/MS

In total, 61 polyphenols were characterized in passion fruit samples, categorized as phenolic acid (19), flavonoids (33), and other phenolic substances (9) (Table 4).

**TABLE 4:** Qualitative analysis of phenolic compounds in different parts of investigated passion fruit samples using LC‐ESI‐QTOF‐MS/MS.

Polyphenols	Proposed compound	Molecular formula	RT (min)	Mode (ESI+/ESI‐)	Molecular weight	Theoretical (*m*/*z*)	Observed (*m*/*z*)	Error (ppm)	MS^2^ Product ions	Sample
Phenolic acid										
Hydroxybenzoic acids										
1	Paeoniflorin	C_23_H_28_O_11_	3.890	[M‐H]^−^	480.1610	479.1537	479.1542	1	449, 357, 327	SHL
2	4‐Hydroxybenzoic acid 4‐*O*‐glucoside	C_13_H_16_O_8_	4.099	[M‐H]^−^	300.0829	299.0756	299.0759	1	255, 137	SWL, FGL^a^
3	Protocatechuic acid 4‐*O*‐glucoside	C_13_H_16_O_9_	4.601	[M‐H]^−^	316.0779	315.0706	315.0699	−2.2	153	SHL, FGL, MGL^a^
4	Protocatechuic acid	C_7_H_6_O_4_	5.630	[M‐H]^−^	154.0253	153.0180	153.0179	−0.7	109	FGL, MGL, SWL, SHL^a^
5	2‐Hydroxybenzoic acid	C_7_H_6_O_3_	6.622	[M‐H]^−^	138.0312	137.0239	137.0240	0.7	93	SHL, FGL, MGL, MGP, SWL, SWP^a^
6	Benzoic acid	C_7_H_6_O_2_	7.549	[M‐H]^−^	122.0369	121.0296	121.0295	−0.8	77	SHL
7	4‐*O*‐Methylgallic acid	C_8_H_8_O_5_	7.076	[M‐H]^−^	184.0364	183.0291	183.0290	−0.5	170, 142	FGL
Hydroxycinnamic acids										
8	Ferulic acid 4‐*O*‐glucoside	C_16_H_20_O_9_	3.972	[M‐H]^−^	356.1103	355.1030	355.1027	−0.8	193, 178, 149, 134	FGL, MGL, SWL, SHL^a^
9	3‐Caffeoylquinic acid	C_16_H_18_O_9_	4.053	[M‐H]^−^	354.0927	353.0854	353.0853	−0.3	253, 190, 144	SWP, FGP, MGP, SHP^a^
10	1‐Sinapoyl‐2‐feruloylgentiobiose	C_33_H_40_O_18_	4.236	[M‐H]^−^	724.2220	723.2147	723.2140	−1	529, 499	FGL, MGL, SHL^a^
11	Ferulic acid	C_10_H_10_O_4_	4.751	[M‐H]^−^	194.0581	193.0508	193.0509	0.5	178, 149, 134	SWL
12	*p*‐Coumaroyl tartaric acid	C_13_H_12_O_8_	4.881	[M‐H]^−^	296.0539	295.0466	295.0475	3.1	115	MGL
13	4,5‐Dicaffeoylquinic acid	C_25_H_24_O_12_	5.477	[M‐H]^−^	516.1266	515.1193	515.1175	−3.5	353, 335	SWP
14	*m*‐Coumaric acid	C_9_H_8_O_3_	5.653	[M‐H]^−^	164.0460	163.0387	163.0388	0.6	119	MGP
15	5–5′‐Dehydrodiferulic acid	C_20_H_18_O_8_	7.440	[M + H]^+^	386.0988	229.0855	387.1071	0.4	369	FGL
16	Caffeoyl glucose	C_15_H_18_O_9_	8.023	[M‐H]^−^	342.0950	341.0877	341.0884	2.1	179, 161	MGL
17	5‐*p*‐Coumaroylquinic acid	C_16_H_18_O_8_	8.023	[M‐H]^−^	338.0987	337.0914	337.0913	−0.3	265, 173, 162	MGL, FGL, SHL^a^
18	Cinnamic acid	C_9_H_8_O_2_	8.173	[M‐H]^−^	148.0523	147.0450	147.0449	−0.7	103	MGL, FGL, SWL, SHL^a^
19	Caffeic acid	C_9_H_8_O_4_	45.706	[M‐H]^−^	180.0424	179.0351	179.0352	0.6	143, 133	MGL, FGL^a^
Flavonoids										
Anthocyanins										
20	4‐*O*‐Methyldelphinidin 3‐*O*‐D‐glucoside	C_22_H_23_O_12_	6.987	[M + H]^+^ ^b^	479.1189	480.1262	480.1259	−0.6	317, 303, 285, 271	FGL, MGL, FGP^a^
21	Cyanidin 3‐*O*‐(6″‐*p*‐coumaroyl‐glucoside)	C_30_H_27_O_13_	48.191	[M‐H]^−^	595.1427	594.1354	594.1353	−0.2	287	MGL, SHL^a^
Dihydrochalcones										
22	3‐Hydroxyphloretin 2′‐*O*‐glucoside	C_21_H_24_O_11_	6.586	[M‐H]^−^	452.1363	451.1290	451.1294	0.9	289, 273	SWP, MGP^a^
23	Phloridzin	C_21_H_24_O_10_	6.900	[M‐H]^−^ ^b^	436.1378	435.1305	435.1294	−2.5	273	FGL, FGP^a^
Dihydroflavonols										
24	Dihydromyricetin 3‐*O*‐rhamnoside	C_21_H_22_O_12_	3.856	[M‐H]^−^	466.1075	465.1002	465.1004	0.4	301	FGL, MGL, SWL, SHL^a^
25	Dihydroquercetin	C_15_H_12_O_7_	5.226	[M‐H]^−^	304.0608	303.0535	303.0533	−0.7	285, 275, 151	FGL, MGL, SHL^a^
26	Dihydroquercetin 3‐*O*‐rhamnoside	C_21_H_22_O_11_	6.787	[M‐H]^−^	450.1170	449.1097	449.1091	−1.3	303, 285, 179	MGL, FGL^a^
Flavanols										
27	(+)‐Gallocatechin	C_15_H_14_O_7_	4.834	[M‐H]^−^	306.0723	305.0650	305.0648	−0.7	261, 219	SWL, FGL, MGL^a^
28	(−)‐Epicatechin	C_15_H_14_O_6_	6.900	[M‐H]^−^ ^b^	290.0786	289.0713	289.0715	0.7	245, 205, 179	FGL, MGL, SWL, SHL^a^
29	4″‐*O*‐Methylepigallocatechin 3‐*O*‐gallate	C_23_H_20_O_11_	7.505	[M‐H]^−^	472.1050	471.0977	471.0978	0.2	169, 319	FGP, MGP, SWP, SHL, SHP^a^
Flavanones										
30	Narirutin	C_27_H_32_O_14_	4.359	[M‐H]^−^	580.1811	579.1738	579.1766	4.8	271	SWL
31	Naringin 4′‐*O*‐glucoside	C_33_H_42_O_19_	4.705	[M‐H]^−^ ^b^	742.2300	741.2227	741.2217	−1.3	433, 271	MGL, FGL^a^
Flavones										
32	Apigenin 6‐*C*‐glucoside	C_21_H_20_O_10_	4.593	[M‐H]^−^ ^b^	432.1089	431.1016	431.1020	0.9	413, 341, 311	FGL, MGL, SWL, SHL^a^
33	Isorhoifolin	C_27_H_30_O_14_	6.155	[M‐H]^−^ ^b^	578.1628	577.1555	577.1553	−0.3	413, 269	MGL, FGL, SWL, SHL^a^
34	Apigenin 6,8‐di‐*C*‐glucoside	C_27_H_30_O_15_	6.959	[M‐H]^−^ ^b^	594.1578	593.1505	593.1506	0.2	503, 473	FGL, MGL, SHL^a^
35	Nepetin	C_16_H_12_O_7_	42.250	[M‐H]^−^	316.0586	315.0513	315.0513	0	300, 271	FGL
Flavonols										
36	Quercetin 3‐*O*‐rhamnoside	C_21_H_20_O_11_	3.856	[M‐H]^−^ ^b^	448.0975	447.0902	447.0898	−0.9	301	FGL, MGL, SWL, SHL^a^
37	Kaempferol 3‐*O*‐(2″‐rhamnosyl‐galactoside) 7‐*O*‐rhamnoside	C_33_H_40_O_19_	3.865	[M + H]^+^	740.2157	741.2230	741.2217	−1.8	593, 447, 285	FGL
38	Kaempferol 3‐*O*‐glucosyl‐rhamnosyl‐galactoside	C_33_H_40_O_20_	4.944	[M‐H]^−^	756.2123	755.205	755.2062	1.6	285	SHL
39	Quercetin 3′‐*O*‐glucuronide	C_21_H_18_O_13_	5.276	[M‐H]^−^	478.0741	477.0668	477.0664	−0.8	301	SHP
40	Myricetin 3‐*O*‐rhamnoside	C_21_H_20_O_12_	7.134	[M‐H]^−^	464.0949	463.0876	463.0880	0.9	317	MGL, FGL, SWL^a^
41	Myricetin 3‐*O*‐galactoside	C_21_H_20_O_13_	7.178	[M‐H]^−^	480.0920	479.0847	479.0844	−0.6	317	SWL
42	Quercetin 3‐*O*‐rutinoside	C_27_H_30_O_16_	23.199	[M‐H]^−^ ^b^	610.1526	609.1453	609.1455	0.3	447, 285	FGL, MGL^a^
Isoflavonoids										
43	Dalbergin	C_16_H_12_O_4_	3.856	[M‐H]^−^	268.0755	267.0682	267.0693	4.1	252, 224, 180	FGL
44	6″‐*O*‐Malonyldaidzin	C_24_H_22_O_12_	3.914	[M‐H]^−^	502.1106	501.1033	501.1014	−3.8	255	MGL
45	Violanone	C_17_H_16_O_6_	3.963	[M‐H]^−^	316.0959	315.0886	315.0888	0.6	300, 285, 135	MGL, FGL, SWL, SHL^a^
46	2′,7‐Dihydroxy‐4′,5′‐dimethoxyisoflavone	C_17_H_14_O_6_	4.857	[M + H]^+^	314.0794	389.1613	337.0688	−3.1	300, 282	FGP
47	Formononetin 7‐*O*‐glucuronide	C_22_H_20_O_10_	5.406	[M + H]^+^ ^b^	444.1100	301.2384	467.0977	4.6	267, 252	FGL
48	5,6,7,3′,4′‐Pentahydroxyisoflavone	C_15_H_10_O_7_	7.177	[M + H]^+^ ^b^	302.0432	303.0505	303.0501	−1.3	285, 257	FGL, FGP, MGL, SWL, SWP, SHL, SHP^a^
49	6″‐*O*‐Acetylglycitin	C_24_H_24_O_11_	7.924	[M‐H]^−^	488.1359	487.1286	487.1293	1.4	285, 270	MGL
50	Tectoridin	C_22_H_22_O_11_	18.746	[M + H]^+^ ^b^	462.1153	463.1226	463.1226	0	445, 427, 409, 381	FGL
51	2‐Dehydro‐*O*‐desmethylangolensin	C_15_H_12_O_4_	43.675	[M‐H]^−^	256.0734	255.0661	255.0662	0.4	135, 119	FGP, MGP, SWP^a^
52	6″‐*O*‐Acetyldaidzin	C_23_H_22_O_10_	45.613	[M‐H]^−^	458.1202	457.1129	457.1135	1.3	221	FGL, MGL^a^
Other polyphenols										
Lignans										
53	7‐Oxomatairesinol	C_20_H_20_O_7_	27.143	[M + H]^+^	372.1184	373.1257	373.1257	0	358, 343, 328, 325	FGL
Alkylmethoxyphenols										
54	4‐Vinylsyringol	C_15_H_14_O_3_	39.705	[M + H]^+^	242.0926	245.0808	243.0996	−0.4	255, 211, 197	FGL
Curcuminoids										
55	Demethoxycurcumin	C_20_H_18_O_5_	5.089	[M‐H]^−^	338.1168	337.1095	337.1096	0.3	217	FGL
Hydroxybenzaldehydes										
56	Vanillin	C_8_H_8_O_3_	7.178	[M‐H]^−^	152.0478	151.0405	151.0409	2.6	136, 92	SWL
57	Syringaldehyde	C_9_H_10_O_4_	28.450	[M‐H]^−^	182.0575	181.0502	181.0503	0.6	163, 135, 119	MGL, FGL, SWL, SHL^a^
Phenolic terpenes										
58	Carnosic acid	C_20_H_28_O_4_	54.506	[M + H]^+^	332.1970	251.0787	333.2042	1.6	287, 269	FGL
Stilbenes										
59	4‐Hydroxy‐3,5,4′‐trimethoxystilbene	C_17_H_18_O_4_	4.439	[M + H]^+^	286.1191	251.0777	304.1535	3.2	271, 241, 225	FGL
60	Resveratrol 5‐*O*‐glucoside	C_20_H_22_O_8_	5.523	[M‐H]^−^	390.1296	389.1223	389.1226	0.8	227	SWL
61	Trans‐Resveratrol	C_14_H_12_O_3_	5.822	[M‐H]^−^	228.0771	227.0698	227.0697	−0.4	212, 185, 157, 143	SWP

*Note*: Passionfruit samples are abbreviated as follows: FGL, Flamingo Peel; FGP, Flamingo Pulp; MGL; Misty Gem Peel; MGP, Misty Gem Pulp; SWL, Sweetheart Peel; SWL, Sweetheart Pulp; SHL, Panama Peel; SWP, Panama Pulp.

^a^ If a compound was detected in more than one sample, data presented in this table are from the sample with an asterisk.

^b^ Compounds detected in both negative [M‐H]− and positive [M + H]+ ionization modes; though only data from one mode are presented.

#### 3.5.1 Phenolic acid

Nineteen phenolic acids were identified in the passion fruit samples examined in this study, which included hydroxybenzoic acids (7) and hydroxycinnamic acids (12).

##### Hydroxybenzoic acids

Compound 1 was proposed as paeoniflorin based on the observed m/z at 497.1542 in negative mode. This identification was further confirmed by an MS/MS experiment displaying the sequential loss of CH_2_O (30 Da) and benzoic acid (122 Da) (Pan et al., 2020). Paeoniflorin is prevalent in the plant *Paeonia lactiflora*, an essential component of traditional medicine known for its functional compounds (Xin et al., 2019). It exhibits bioactive effects in modulating immune cells and the production of inflammatory mediators, resulting in anti‐inflammatory and immunoregulatory effects (L. Zhang & Wei, 2020).

Two derivatives of hydroxybenzoic acids were identified: Compounds 5 and 2. Compound 5, 2‐hydroxybenzoic acid (*m*/*z* 137.0240), exhibited a product ion by losing CO_2_ (44 Da) at *m*/*z* 93 in negative mode (Shi et al., 2021). Compound 2 (*m*/*z* 299.0759) was identified as 4‐hydroxybenzoic acid‐4‐*O*‐glucoside, presenting product ions at m/z 255 and *m*/*z* 137, indicating the loss of CO_2_ (44 Da) and glucoside (162 Da) from the precursor, respectively (Shi et al., 2021). 4‐Hydroxybenzoic acid, also known as p‐hydroxybenzoic acid, was found abundantly in purple passion fruit peel, with a content of 2.1 mg/g in previous studies (Suleria et al., 2020). Compounds 3 (*m*/*z* 315.0699) and 4 (*m*/*z* 153.0179), identified exclusively in peel samples, were suggested to be protocatechuic acid‐4‐*O*‐glucoside and protocatechuic acid, respectively.

##### Hydroxycinnamic acids

Compounds 8 and 18, ferulic acid 4‐*O*‐glucoside (*m*/*z* 355.1030) and cinnamic acid (*m*/*z* 147.0449), were detected in all peel samples. In the MS^2^ experiment, cinnamic acid displayed product ions at *m*/*z* 103, indicating the loss of CO_2_ (44 Da). Ferulic acid‐4‐*O*‐glucoside exhibited fragments at *m*/*z* 197, *m*/*z* 178, *m*/*z* 149, and *m*/*z* 134, signifying the disintegration of ferulic acid followed by sequential loss of CH_3_, H_2_O, and CH_3_ with CO_2_ (Suleria et al., 2020). Compound 11, ferulic acid, also produced the same fragments. This observation aligns with prior studies that found ferulic acid exclusively in the peel of yellow and purple passion fruit (Purohit et al., 2021; Wang et al., 2015). Ferulic acid and its glucoside have been extensively studied in traditional medicine for health benefits such as improving lipid profiles, reducing tumor weight, and protecting against liver injury (Wang et al., 2015). Similarly, compound 19 (m/z 179.0352), detected in peel samples MGL and FGL, was proposed as caffeic acid, indicating the loss of H_2_O and HCOOH (46 Da) at [M‐H]^−^ *m*/*z* 143 and 133 (Wang et al., 2015).

Meanwhile, phenolic acids like compounds 9, 13, and 14 were only detected in pulp samples. Compounds 9 (*m*/*z* 353.0853) and 13 (*m*/*z* 515.1175) were proposed as 3‐caffeoylquinic acid and 4,5‐dicaffeoylquinic acid, respectively. The observed fragment at *m*/*z* 353 in the MS^2^ experiment of compound 13 (4,5‐dicaffeoylquinic acid) was presumably produced by the loss of caffeic acid. 3‐Caffeoylquinic acid, also known as chlorogenic acid, is commonly found in coffee and tea. Several studies have successfully quantified high levels of chlorogenic acid in fruits and by‐products, including apples, mangoes, plums, quinces, and sweet cherries (Meinhart et al., 2019; Suleria et al., 2020). Leite et al. (2021) and Wang et al. (2015) did not detect chlorogenic acid in passion fruits and their mixed beverages; instead, they found high content of its isomers, such as 4‐caffeoylquinic acid and 5‐caffeoylquinic acid. Chlorogenic acid was previously associated with the repression of carcinogenic cells, modulation of skeletal muscle glucose, and improvement of lipid profiles (Meinhart et al., 2019). Compound 14 (*m*/*z* 163.0388) was tentatively identified as *m*‐coumaric acid based on the [M‐H]^−^ at *m*/*z* 119 (Suleria et al., 2020). Phenolic *p*‐coumaric acid serves as the precursor of chlorogenic acid and other phenolic acids. Notably, only *m*‐coumaric acid was observed in MGP samples, in contrast to previous studies that reported a high content of p‐coumaric acid (Corrêa et al., 2016). This difference can be attributed to variations in plantation conditions, genotypes, and extraction methods.

#### 3.5.2 Flavonoids

##### Flavanols

Initially, three catechin derivatives were identified. Compound 27 (*m*/*z* 289.0715) and compound 28 (*m*/*z* 289.0715) were proposed as (+)‐gallocatechin and (−)‐epicatechin, respectively, and were further confirmed by the MS^2^ experiment. Previously, (−)‐epicatechin was characterized in several passion fruit varieties, including *P*. *edulis* var. *Flavicarp*, *P*. *edulis* var. *Sims*, and *P*. *ligularis* var. *Juss* (Carmona‐Hernandez et al., 2019). These two flavanols were widely identified in the peel of *P*. *edulis* and extensively studied for their health benefits, particularly their antioxidant and anticancer potential (Dominguez‐Rodriguez et al., 2019; Zibadi et al., 2007). Compound 29 (*m*/*z* 471.0978) was observed in all *P*. *edulis* samples and tentatively characterized as 4″‐*O*‐methylepigallocatechin‐3‐*O*‐gallate in negative mode. (−)‐Epigallocatechin‐3‐*O*‐gallate (EGCG) is abundant in tea and recognized as one of the most essential catechins in tea phenolic compounds (Kirita et al., 2014). Flavonoid EGCG has previously been detected in different passion fruit varieties. However, this is the first instance where the O‐methylated form of EGCG was identified in passion fruit. This finding is noteworthy for its potential to enhance bioavailability and the efficacy of bioactivity (Oritani et al., 2013).

##### Flavanones

Two flavanone glucosides were detected in the studied passion fruit peel samples. Compounds 30 and 31, observed at *m*/*z* 579.1738 and 741.2217, respectively, were proposed as narirutin and naringin 4′‐*O*‐glucoside, a proposal further confirmed by the presence of observed fragments naringenin (*m*/*z* 271) and naringenin 4′‐*O*‐glucoside (*m*/*z* 433) (Zhang & Brodbelt, 2004). Narirutin is abundantly found in citrus fruit peels and is responsible for several preventive effects against chronic disease (Xi et al., 2015). A recent study has further highlighted the potential positive impact of narirutin in preventing prostate cancer by modulating cell life cycles and binding with hyaluronidase (Singh et al., 2023). The identification of narirutin offers evidence for further studies related to the health benefits of Australian passion fruits.

##### Flavones

Three flavone glucosides and a methylated flavone were identified in this study. Focusing on the observed *m*/*z* values at 431.1020, *m*/*z* 577.1553, and *m*/*z* 593.1506 in both negative and positive modes, compound 32, compound 33, and compound 34 were identified as apigenin 6‐c‐glucoside, isorhoifolin, and apigenin 6,8‐di‐C‐glucoside, respectively, with several characteristic ions in the MS^2^ experiment. Passion fruits are rich sources of C‐glucoside phenolic compounds. Among them, vitexin and orientin are two widely studied flavone glucosides known for their strong antioxidant capacity and their ability to modulate gene expression related to inflammation (Benincá et al., 2007; do Carmo Santos et al., 2021; Saravanan et al., 2014). In this study, flavones were only detected in peel samples, which aligns with previous research findings. However, neither luteolin nor its glucoside were detected in Australian passion fruit samples, which could be attributed to variations in genotype, plantation conditions, and maturity levels (do Carmo Santos et al., 2021).

##### Flavonols

The flavonols identified in the studied passion fruit samples consist of the glucosides quercetin, kaempferol, and myricetin. Quercetin, kaempferol, and myricetin share the same structure, with characteristic differences in terms of the hydroxy group positions in the C‐ring. Compounds 37 (*m*/*z* 741.2217) and 38 (*m*/*z* 755.2062), present in FGL and SHL samples, were characterized as Kaempferol 3‐*O*‐(2″‐rhamnosyl‐galactoside) and Kaempferol 3‐*O*‐glucosyl‐rhamnosyl‐galactoside, respectively, with their observed ions in the MS^2^ experiment at *m*/*z* 285 indicating the breakdown of kaempferol. A similar fragment in the MS^2^ experiment was also observed in compounds 40 and 41, both of which were myricetin glucosides with the same observed ion at *m*/*z* 301, indicating the breakdown of myricetin. In this study, flavonol glucosides were mainly detected in the peel samples, except for compound 39 (*m*/*z* 301), which is quercetin glucuronide, present in the SHP sample.

##### Anthocyanins

4‐*O*‐Methyldelphinidin 3‐*O*‐D‐glucoside was identified in FGP, FGL, and MGL at 480.1259 *m*/*z* in negative mode. To our knowledge, this is the first time this anthocyanin has been identified in passion fruits. It was previously identified in bamboo seed rice. Cyanidin 3‐*O*‐(6″‐*p*‐coumaroyl‐glucoside) was found in FGL and MGL at 594.1353 *m*/*z* in negative mode. This anthocyanin was also discovered in blackberries in previous studies.

##### Dihydrochalcones and dihydroflavonols

Five compounds were found in dihydrochalcones and dihydroflavonols. All three identified dihydroflavonols were exclusively found in the peel samples. Dihydromyricetin 3‐*O*‐rhamnoside was identified in the peel of all four studied varieties, while dihydroquercetin was only identified in three *P*. *edulis* varieties (FGL, MGL, and SHL). The glucoside of dihydroquercetin was also identified as compound 26 in MGL and FGL. Phloridzin was only identified in the FGL and FGP. This result is consistent with a previous study where phloridzin was identified in ethanol extracts of *P*. *edulis* peel (L. C. Dos Santos et al., 2021).

#### 3.5.3 Other polyphenols

In addition to the phenolic acids and flavonoids mentioned earlier, several different polyphenols were observed in this study, including lignans (1), alkylmethoxynhenols (1), curcuminoids (1), hydroxybenzaldehydes (2), phenolic terpenes (1), and stilbenes (3).

Compound 56 (*m*/*z* 151.0409) and compound 57 (*m*/*z* 151.0503) were tentatively identified as vanillin and syringaldehyde, found only in the peel samples of passion fruits. These hydroxybenzaldehyde compounds were previously reported as essential aroma compounds developed during wine aging, contributing to unique aroma and color. Additionally, hydroxybenzaldehydes are significant precursors of resveratrol in plant metabolites (Davis et al., 2013).

Stilbenes extracted from passion fruits have been widely studied for their potential anti‐cancer and various health benefits (Arai et al., 2016; Dos Santos et al., 2022; Muzzio et al., 2012). In this study, three stilbenes were identified. Compound 61 (*m*/*z* 227.0697) was proposed as trans‐resveratrol and further confirmed by observed fragments at *m*/*z* 185 and *m*/*z* 143 in negative mode, indicating the successive loss of C_2_H_2_O (42 Da), in alignment with a previous study by Suleria et al. (2020). Compound 60 (*m*/*z* 389.1226) was characterized as resveratrol 5‐*O*‐glucoside with observed fragments in negative mode at *m*/*z* 227, indicating the breakdown of resveratrol (Muzzio et al., 2012). A similar fragment was observed in compound 59 (*m*/*z* 304.1535) in positive mode at *m*/*z* 205, further confirmed as 4‐hydroxy‐3,5,4′‐trimethoxystilbene. Methylated stilbenes like 4‐hydroxy‐3,5,4′‐trimethoxystilbene were previously found in methanol extracts of traditional medicine, *Dendrobium gratiosissimum*, exhibiting moderate inhibitory activity against HIV and IAV viruses (Jia‐Wei et al., 2020). Previous studies have shown the abundance of resveratrol and piceatannol in passion fruit seeds and pulp (Barbosa Santos et al., 2021; Lourencao Zomer et al., 2021). However, in contrast to previous findings, piceatannol was not detected in this study. This difference could be attributed to stilbene instability and variations in extraction parameters.

### 3.6 Distribution of polyphenols – Venn diagram analysis

**FIGURE 3:**
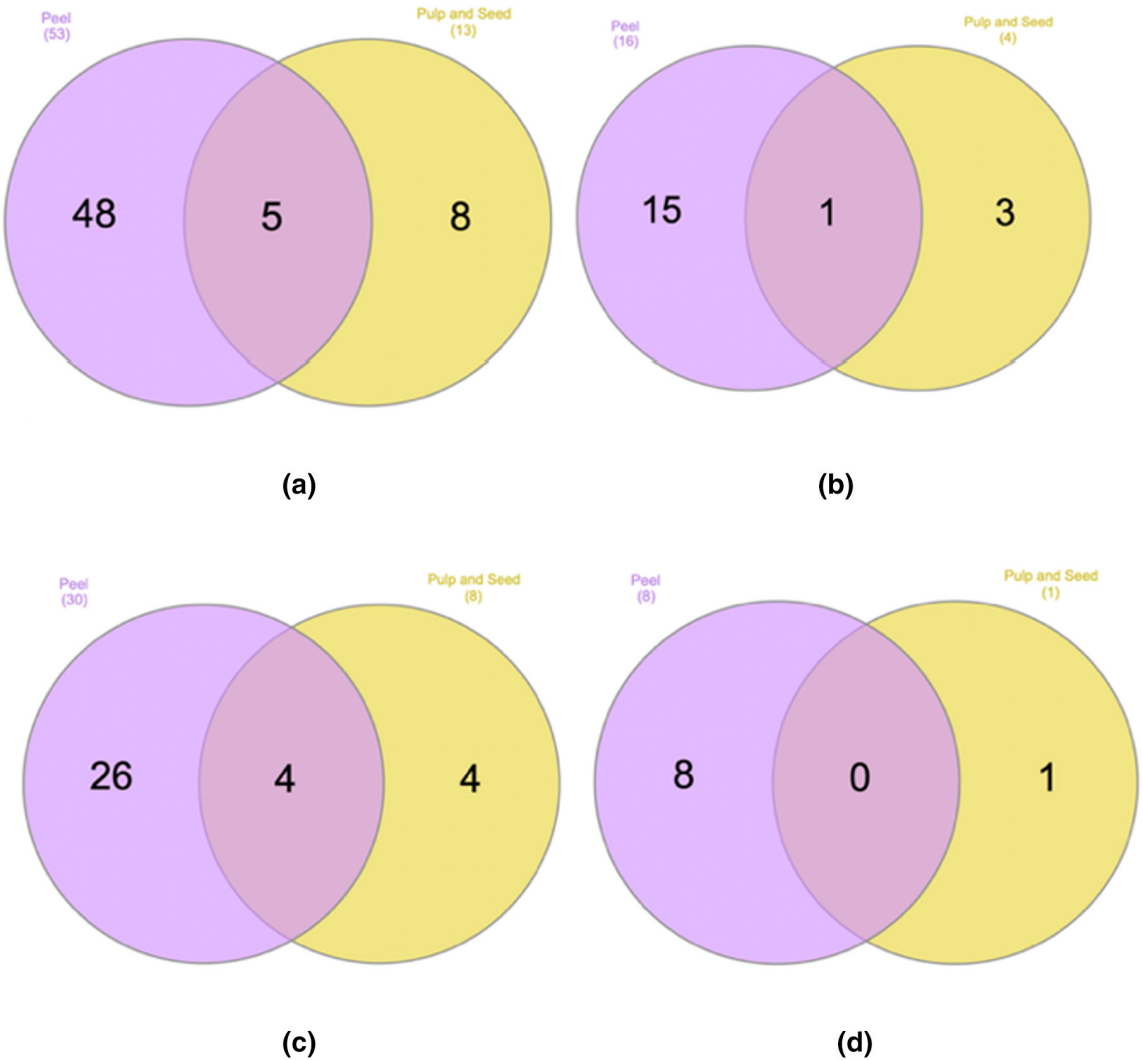
Venn diagram showcasing the distribution of characterized polyphenols between by‐products and edible parts. (a) Total polyphenols; (b) phenolic acid polyphenols; (c) flavonoids; (d) other polyphenols.

**FIGURE 4:**
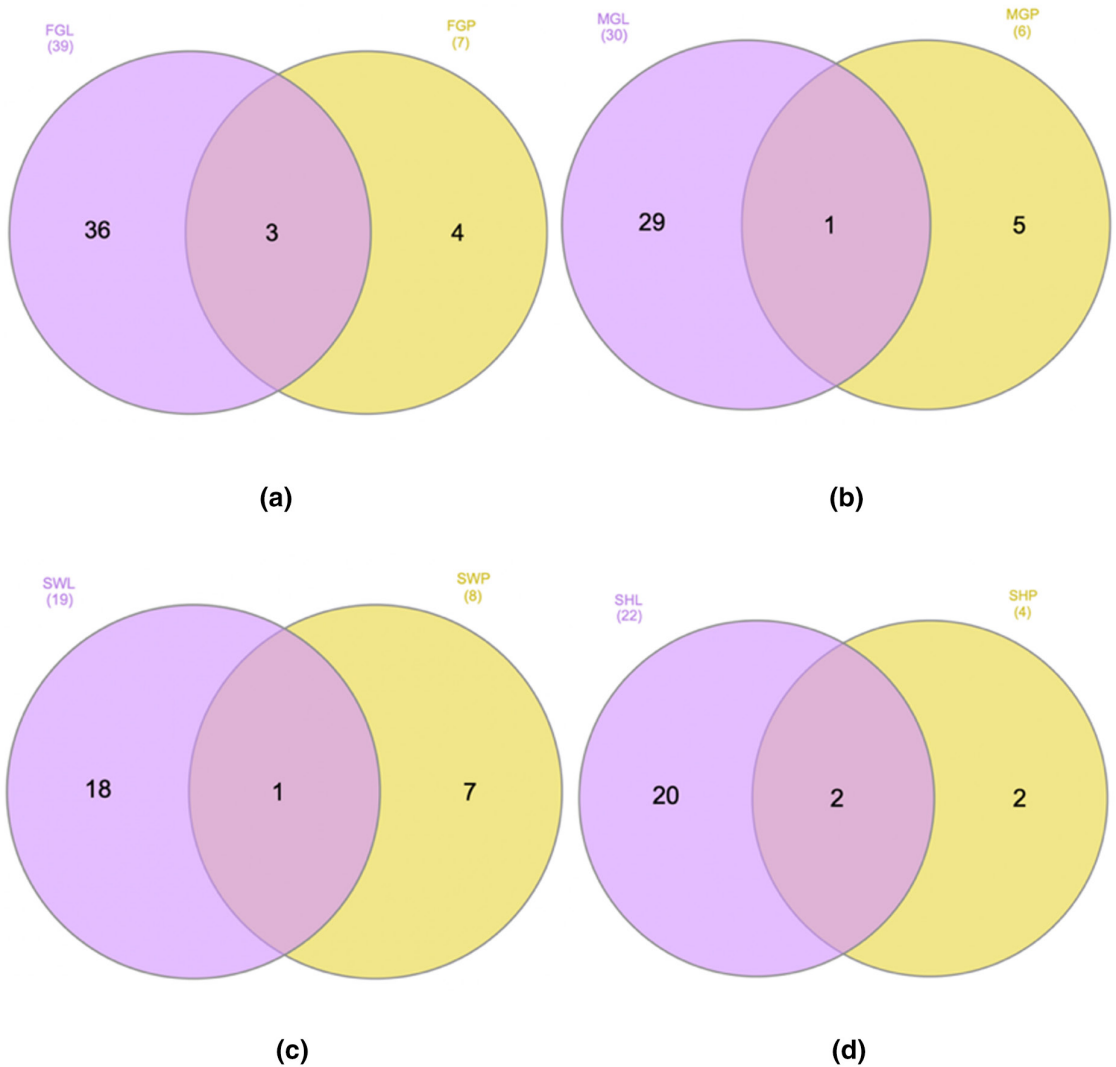
Venn diagram depicting the distribution of characterized polyphenols between by‐products and edible parts in different cultivars. (a) Polyphenols in Flamingo; (b) polyphenols in Misty Gem; (c) polyphenols in Sweetheart; and (d) polyphenols in Panama.

**FIGURE 5:**
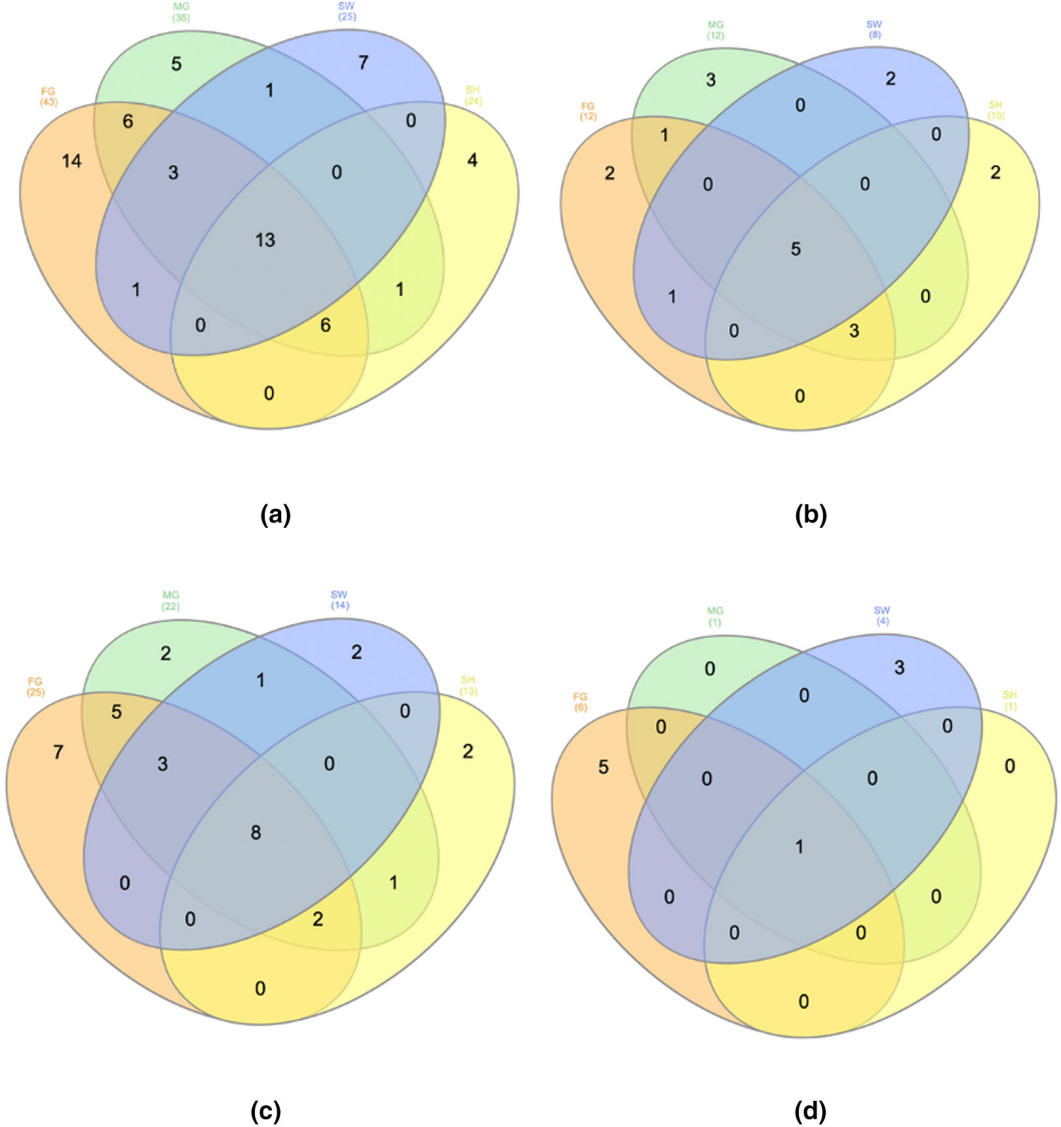
Venn diagram highlighting the distribution of characterized polyphenols among different cultivars. (a) Total polyphenols; (b) phenolic acid polyphenols; (c) flavonoids; (d) other polyphenols.

Using LC‐ESI‐QTOF‐MS/MS, we characterized the polyphenol composition as detailed previously. To gain a deeper insight into the distribution of polyphenols in passion fruit, we employed Venn diagrams (Figures 3, 4, 5) to elucidate the differences across cultivars and portions based on the approach by (Heberle et al., 2015).

Figure 2 contrasts the polyphenol profiles of the by‐product and edible portions. Among the 61 characterized polyphenols, a remarkable 79% were exclusive to the peel, whereas a mere 13% were unique to the edible part. A similar trend was observed across all cultivars, as depicted in Figure 3. Furthermore, the peel samples exhibited a broader spectrum of polyphenols, particularly in terms of phenolic acids, flavonoids, and other phenolic compounds. This diversity in polyphenolic content aligned with our findings, which demonstrated a heightened phenolic content and antioxidant capacity in peel samples.

In terms of polyphenol distribution among the cultivars, FG emerged as the most diverse, boasting 43 identified polyphenols, as illustrated in Figure 4. In contrast, SW and SH displayed the least diversity, with only 25 and 24 polyphenols characterized, respectively. Similar disparities were noted for phenolic acids and flavonoids. For other polyphenolic compounds, while FG exhibited the most diverse profile, SW was identified as a significant source of these polyphenols. Thirteen polyphenols were consistently found across all cultivars, comprising 5 phenolic acids, 8 flavonoids, and a single other polyphenolic compound. Each cultivar also harbored unique polyphenols, which can be attributed to factors related to genotype and cultivation.

## 4 CONCLUSIONS

This study elucidated the phenolic content and antioxidant potential of various sections and cultivars of Australian native passion fruits using in vitro assays. A notable observation is the significantly higher phenolic content in the by‐product parts of the passion fruit, which corresponded to an enhanced antioxidant capacity in these portions, as evidenced by the DPPH, ABTS, FRAP, RPA, and PMA assays. Among the cultivars examined, FG and MG stood out with the most abundant phenolic content and superior antioxidant properties.

Employing LC‐ESI‐QTOF‐MS/MS, we successfully qualified the polyphenols in the Australian native passion fruit, identifying a total of 61 polyphenols. Significantly, some of these polyphenols have been previously recognized for their potential health benefits. Our investigation, further visualized through a Venn diagram, unveiled the distribution of these characterized polyphenols, showing that the peel samples boasted a richer diversity of polyphenols. Moreover, FG was distinctly identified for its exceptional diversity in phenolic acids, flavonoids, and other polyphenolic compounds.

In essence, Australian native passion fruits demonstrate a rich phenolic content and potent in vitro antioxidant capacity, suggesting promising avenues for pharmaceutical applications and underscoring the potential health benefits of these fruits.

## AUTHOR CONTRIBUTIONS

**Haoyao Liu:** Conceptualization (equal); formal analysis (lead); investigation (lead); methodology (lead); validation (lead); writing – original draft (lead). **Osman Tuncay Agar:** Conceptualization (equal); formal analysis (supporting); investigation (supporting); methodology (equal); supervision (equal); validation (supporting); writing – original draft (supporting); writing – review and editing (lead). **Ali Imran:** Supervision (equal); writing – review and editing (supporting). **Colin J. Barrow:** Resources (supporting); supervision (supporting). **Frank R. Dunshea:** Resources (supporting); supervision (supporting). **Hafiz A. R. Suleria:** Conceptualization (equal); formal analysis (supporting); funding acquisition (lead); investigation (equal); methodology (equal); resources (lead); supervision (lead); validation (equal); writing – original draft (equal); writing – review and editing (equal).

## FUNDING INFORMATION

Hafiz Suleria has been honored with the Australian Research Council—Discovery Early Career Award (ARC‐DECRA—DE220100055), backed by the Australian Government. The University of Melbourne has provided funding for this work through the McKenzie Postdoctoral Fellowship Program (grant reference UoM‐18/21), along with contributions from the Future Food Hallmark Research Initiative (grant reference UoM‐21/23) and a Collaborative Research Development Grant (also grant reference UoM‐21/23), both of which are endorsed by the University's Faculty of Science.

## CONFLICT OF INTEREST STATEMENT

The authors declare no potential conflict of interest.

## Data Availability

The data that support the findings of this study are available on request from the corresponding author.
